# Supplementary material for: Control of odor sensation by light and cryptochrome in the Drosophila antenna
Source: iScience. 2025 Apr 16;28(5):112443. doi: 10.1016/j.isci.2025.112443 (PMC12090271; doi:10.1016/j.isci.2025.112443)
Supplement: Document S1. Figures S1–S7 [file mmc1.pdf]

iScience, Volume *28*

Supplemental information

Control of odor sensation by light

and cryptochrome in the *Drosophila* antenna

Dhananjay Thakur, Sydney Hunt, Tiffany Tsou, Miles Petty, Jason M. Rodriguez, and Craig Montell

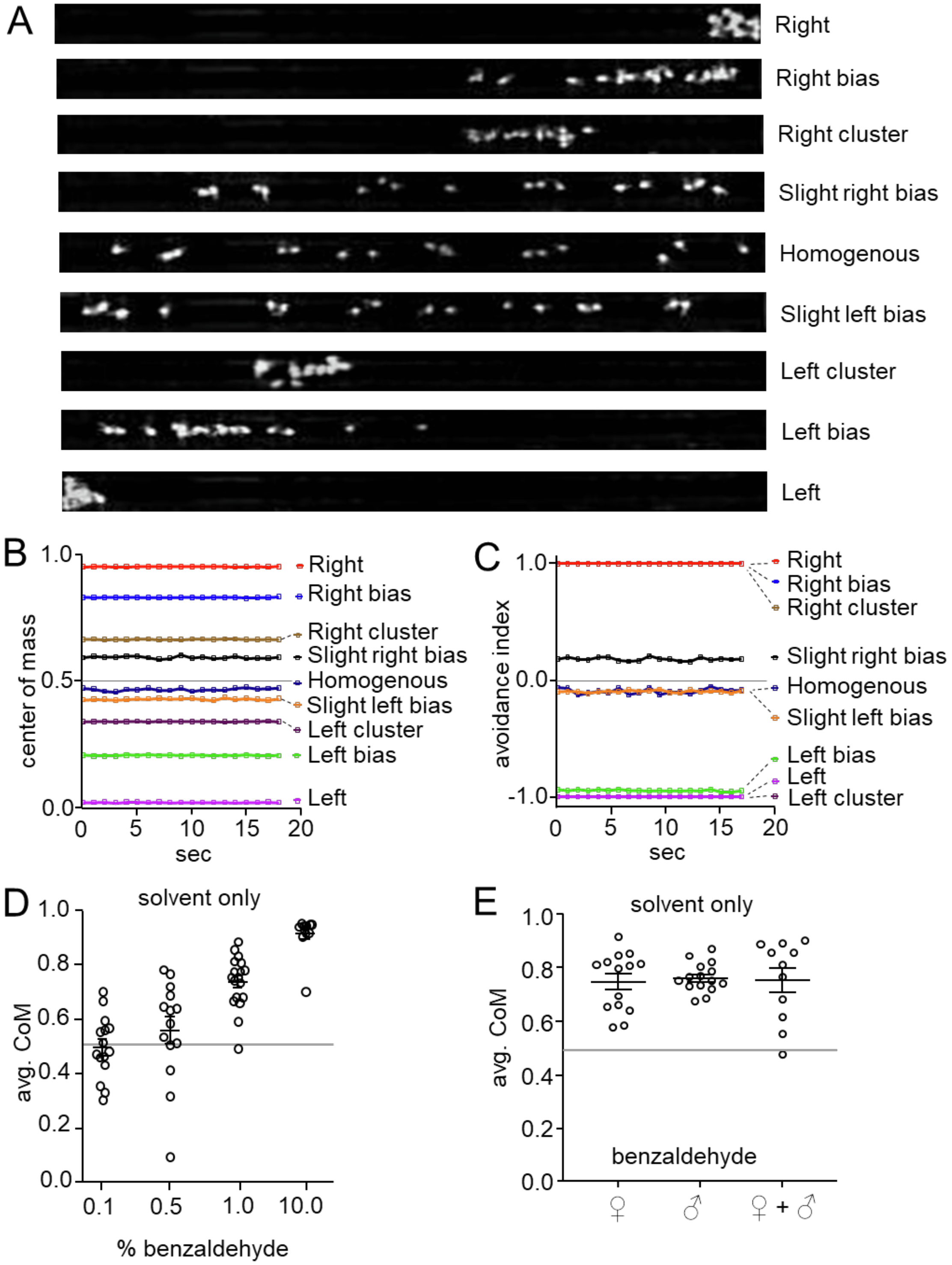

Figure S1. Design and calibration of the experimental paradigm for the DART2 odor assay, Related to Figure 1.

(A) Images of experimental tubes carrying flies immobilized by CO_2 and arranged in 9 different positions simulating different outcomes observed in DART2 assays.
(B) Tubes shown in (A) were analyzed using CoM as the measure of fly positions. See the Methods section (DART2 assays) for the CoM formula.
(C) Tubes shown in (A) were analyzed using the avoidance index as the measure of fly positions. See the Methods section (DART2 assays) for the avoidance index formula.
(D) Average CoM of control flies in response to varying concentrations of BA from 20–60 min of the duration of the assay under N-IR illumination only.
(E) Flies do not show any sexual dimorphism in the two-way assay. Average CoM of control flies in response to 1% BA. Assay tubes contained males, females, or both. n = 11 – 15. ANOVA followed by Dunn's multiple comparisons test. Error bars indicate means ± SEMs. Differences were not significant.

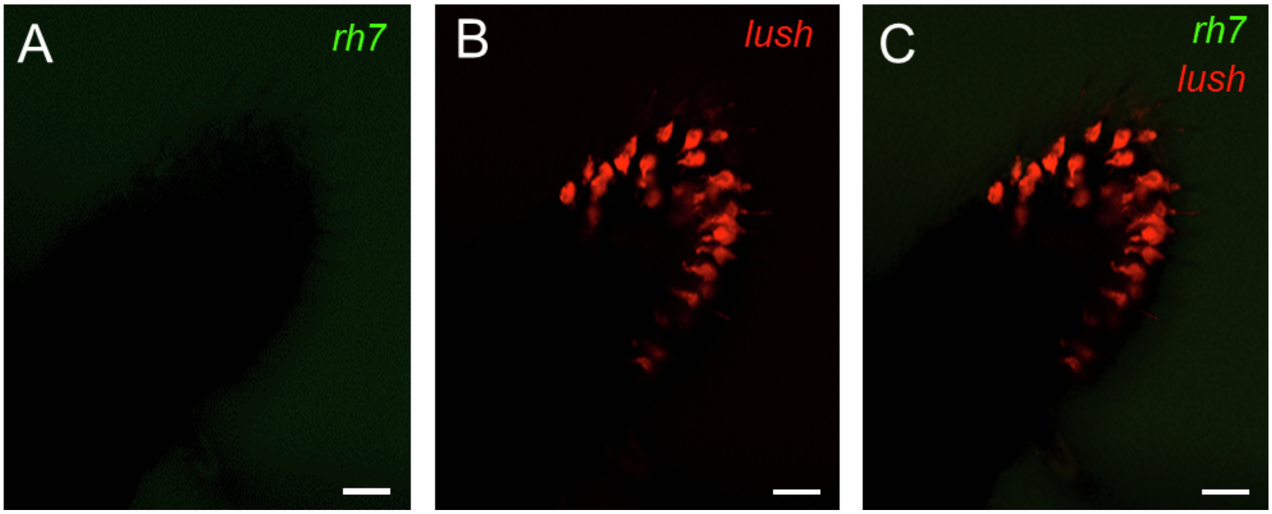

Figure S2. Testing for *rh7* reporter expression in the antenna, Related to Figure 3.
(A) Cross-section of antenna indicating lack of *lexAop-mCD8:GFP* expression driven by the *rh7-LexA*. The antenna was stained with anti-GFP.
(B) Same antenna as (A) showing *UAS-mCD8::RFP* driven by the *lush-GAL4* and stained with anti-RFP.
(C) Overlay of (A) and (B).
The length of each scale bar is 20 μm.

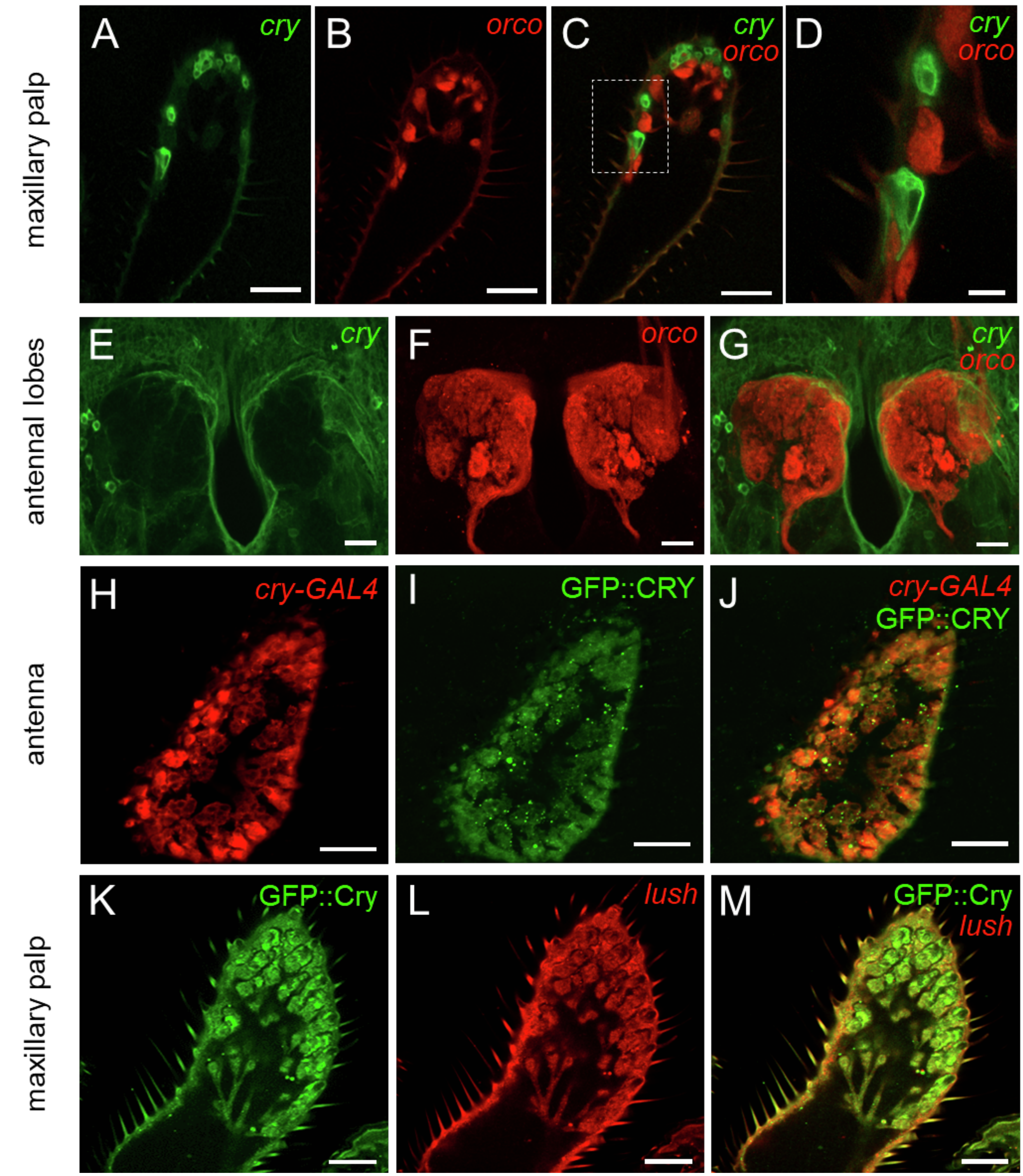

Figure S3. Expression of the *cry*, *orco* and *lush* reporters in olfactory organs, Related to Figure 4.

(A) Cross-section of maxillary palp showing *UAS-mCD8::GFP* expression driven by the *cry-GAL4* and stained with anti-GFP.

(B) Same sample as (A) showing expression of *RFP* driven directly by the *orco* promoter (*orco-RFP*) and stained with anti-RFP.
(C) Merge of (A) and (B). In (A), (B), and (C) the length of each scale bar is 20 μm.
(D) Magnified section marked in (C) by white dotted rectangle. The length of the scale bar is 5 μm.
(E) Z-stack of antennal lobe showing *UAS-mCD8::GFP* expression driven by the *cry-GAL4* and stained with anti-GFP.
(F) Same sample as (E) showing *RFP* driven directly by the *orco* promoter (*orco-RFP*) and stained with an anti-RFP.
(G) Merge of (E) and (F). In (E), (F), and (G) the length of each scale bar is 20 μm.
(H) Antennal cross-section showing expression of *UAS-mCD8::DsRed* driven by the *cry-GAL4* and stained with anti-DsRed.
(I) Same sample as (H) showing expression of GFP-tagged Cry (GFP::Cry) stained with anti-GFP.
(J) Merge of (H) and (I). In (H), (I), and (J) the length of each scale bar is 20 μm.
(K) Maxillary palp cross-section showing expression of GFP::Cry stained with anti-GFP.
(L) Same sample as (K) showing expression of *UAS-mCD8::DsRed* driven by the *lush-GAL4* and stained with anti-DsRed.
(M) Merge of (K) and (L). In (K), (L), and (M) the length of each scale bar is 20 μm.

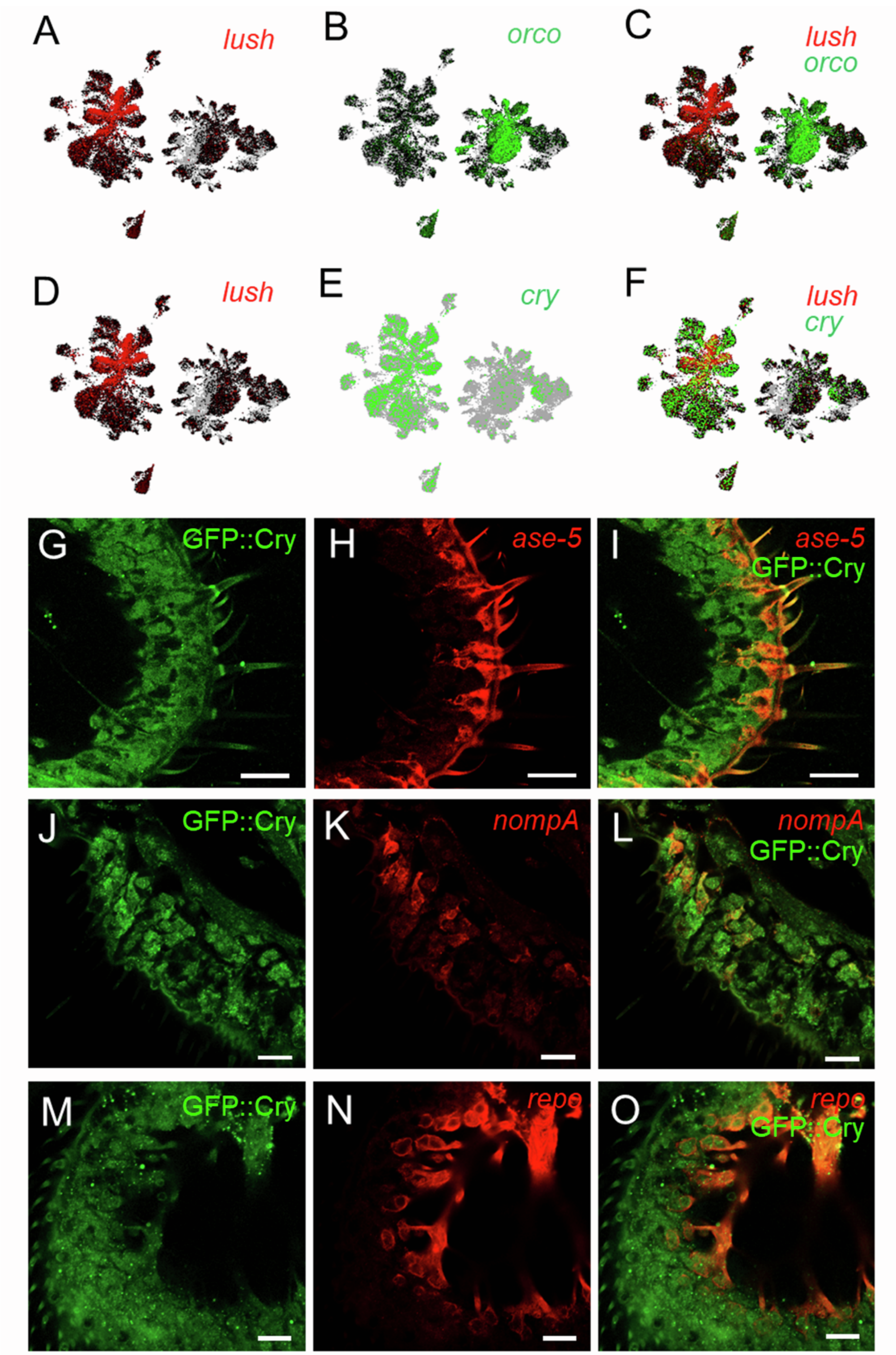

Figure S4. Abundance of *cry* transcripts in the antennae, and testing for expression overlap in the antenna between the GFP::Cry reporter and support cell-type reporters, Related to Figure 4.

(A-F) mRNA transcript levels plotted using FlyCellAtlas (https://flycellatlas.org/) and visualized using the Scope platform with the following method: SmartSeq2> Antenna> Stringent> s_fca_biohub_antenna_10X_ss2. (A) *lush* (red). (B) *orco* (green). (C) Merge of (A) and (B). (D) *lush* (red). (E) *cry* (green). (F) Merge of (D) and (E).
(G) Cross-section showing expression of GFP::Cry stained with anti-GFP.
(H) Same sample as (G) showing expression of *UAS-mCD8::DsRed* driven by the *ase5-GAL4* and stained with anti-DsRed.
(I) Merge of (G) and (H).
(J) Cross-section showing expression of GFP::Cry stained with anti-GFP.
(K) Same sample as (J) showing expression of *UAS-mCD8::DsRed* driven by the *nompA-GAL4* and stained with anti-DsRed .
(L) Merge of (J) and (K).
(M) Cross-section showing expression of GFP::Cry stained with anti-GFP.
(N) Same sample as (M) showing expression of *UAS-mCD8::DsRed* driven by the *repo-GAL4* and stained with anti-DsRed.
(O) Overlay of (M) and (N).
The length of each scale bar is 10 μm.

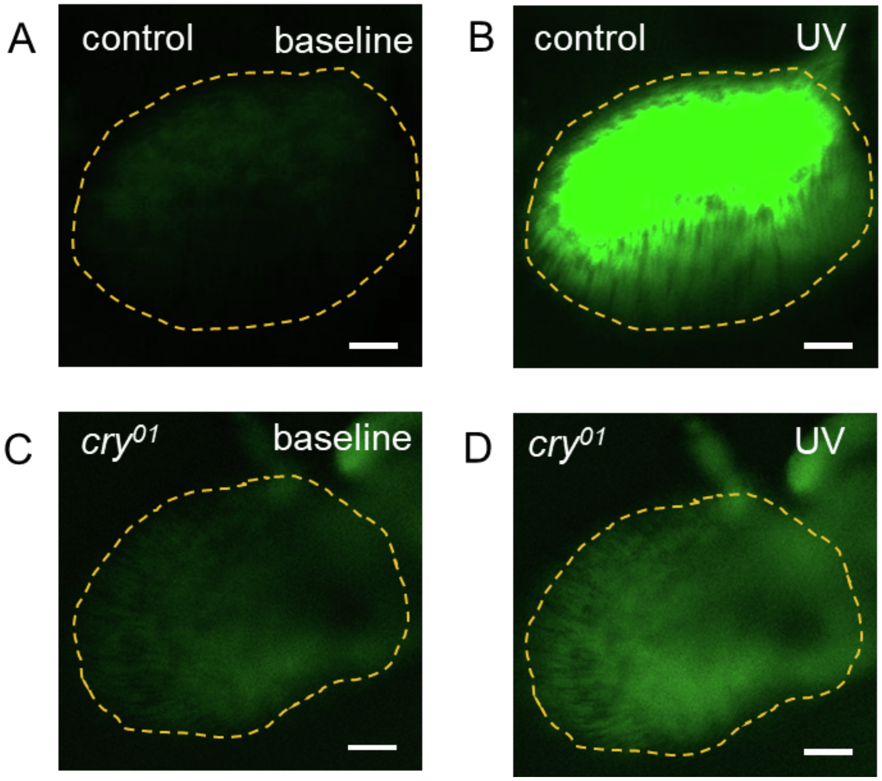

Figure S5. ROS generation in isolated antennae under UV stimulation, Related to Figure 5.
(A) Dissected antenna from control flies incubated with DCFDA and imaged without UV stimulation. Dotted boundary indicates the region of interest used to determine the total DCFDA signal summarized in Figure 5B.
(B) Same antenna as shown in (A) after stimulation with UV light (365 nm) for 1 second.
(C) Dissected antenna from cry^{01} flies incubated with DCFDA.
(D) Same antenna as shown in (C) after stimulation with UV light (365 nm) for 1 second.
The length of each scale bar is 20 μm.

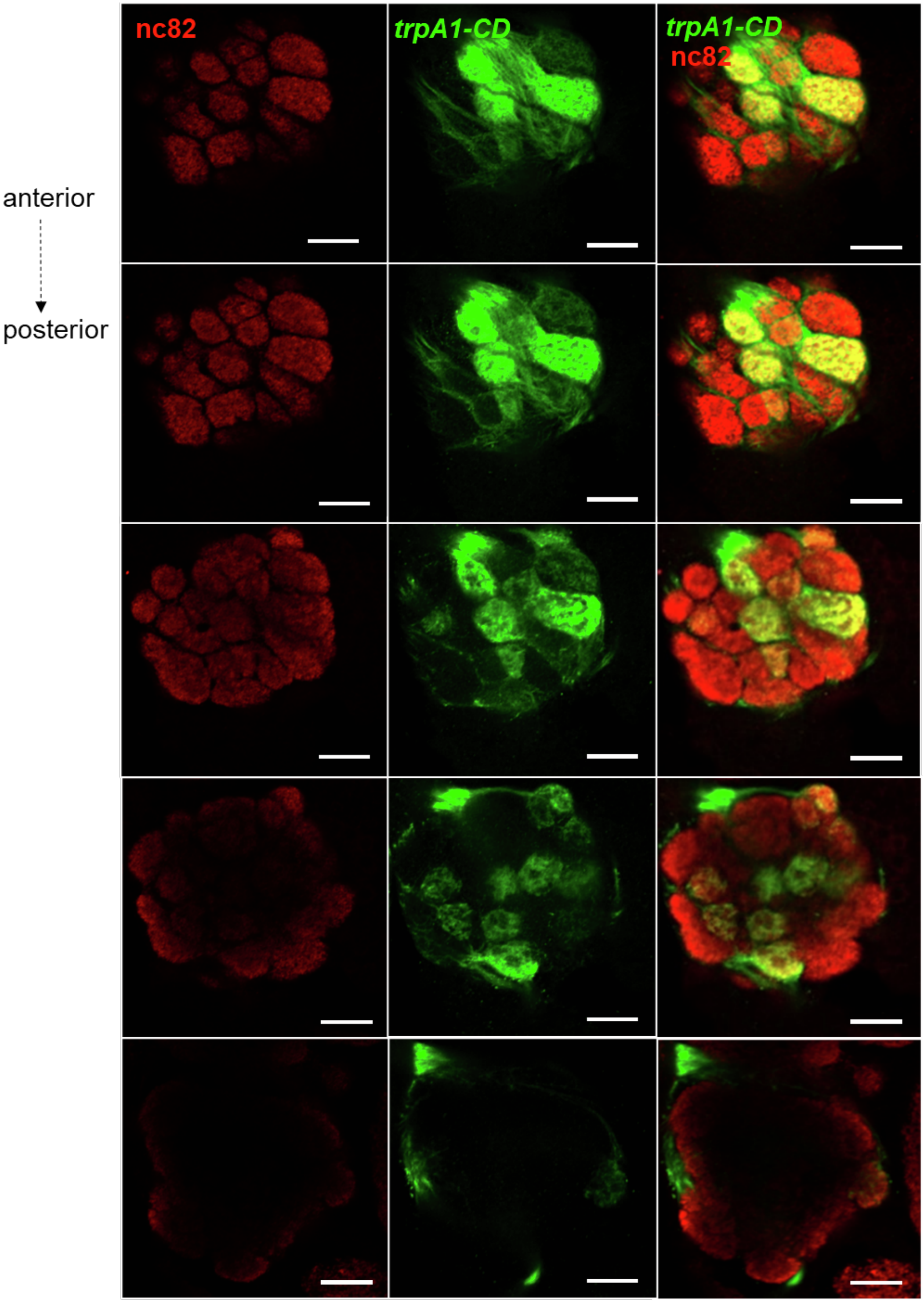

Figure S6. Cross sections of the antennal lobe showing *trpA1-CD* ORN processes, Related to Figure 6. *UAS-mCD8::GFP* was driven by the *trpA1-CD-GAL4* and stained with anti-GFP (green). The tissue was also stained with anti-nc82. The distance between every section and the previous one, starting from the second section was 1, 3, 5 and 7 μm, respectively. The length of each scale bar is 20 μm.

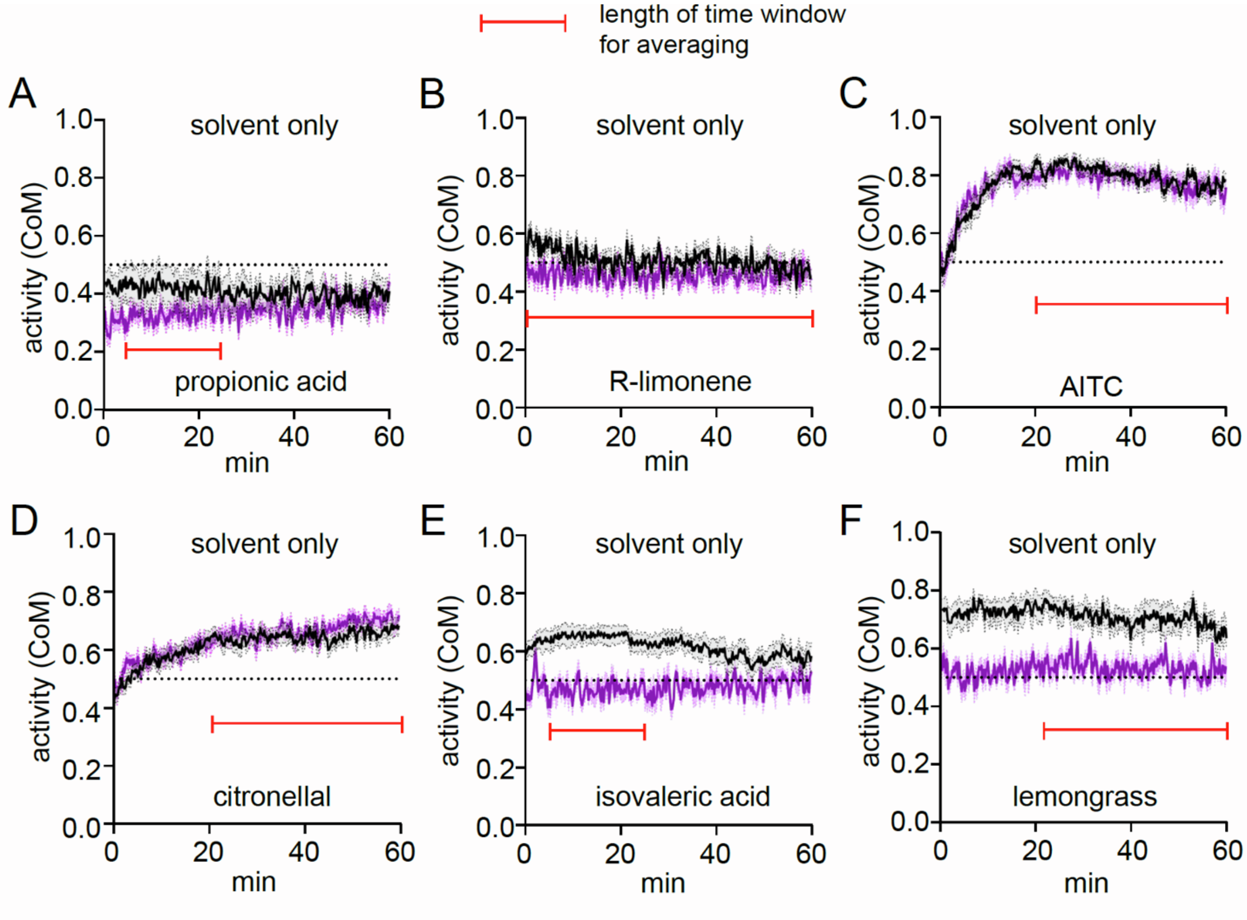

Figure S7. Time courses for behavior responses to odorants in the DART2 assay, Related to Methods. The various odorants used are indicated below. The time-windows that were used to calculate the average CoM are indicated by the red bracketed lines.

(A) 1% propionic acid,

(B) 1% R-limonene,

(C) 0.1% AITC,

(D) 3% citronellal,

(E) 0.01% isovaleric acid,

(F) 3% lemongrass.
